# Risk of Parkinson's disease and depression severity in different populations: A two‐sample Mendelian randomization analysis

**DOI:** 10.1002/brb3.3642

**Published:** 2024-09-01

**Authors:** Yidan Qin, Jia Li, Wei Quan, Jia Song, Jing Xu, Jiajun Chen

**Affiliations:** ^1^ Department of Neurology China‐Japan Union Hospital of Jilin University Changchun China

**Keywords:** ever depressed for a whole week, major depressive disorder, Mendelian randomization, Parkinson's disease

## Abstract

**Background:**

Depression is widely recognized as a common non‐motor symptom of Parkinson's disease (PD). Across different studies, the reported prevalence of depression in PD varies widely, ranging from 2.7% to 90%, but it is unclear whether this association is due to genetic or acquired factors. Whether there is a causal relationship remains unknown. The aim of this study was to use a two‐sample Mendelian randomization (MR) approach to investigate the causal effect of PD on depression.

**Methods:**

Analyses were conducted separately for individuals of European and East Asian ancestry using publicly available summary data from genome‐wide association studies. Depression was divided into two categories: ever depressed for a whole week and major depressive disorder (MDD). PD data were used as the exposure and were obtained from the International Parkinson's Disease Genomics Consortium and the BioBank Japan PheWeb, while depression data were used as the outcome and were obtained from the ntegrative Epidemiology Unit (IEU) Open GWAS Project(A public GWAS database） and the Psychiatric Genomics Consortium. The influence of PD on depression was assessed using inverse variance weighted (IVW), weighted median, MR‐Egger, and weighted mode methods. Heterogeneity and pleiotropy were tested, and the results were validated using FinnGen GWAS data from version R9.

**Results:**

In individuals of European ancestry, there was a causal relationship between PD and ever depressed for a whole week (IVW method, odds ratio [OR] = 0.990; 95% CI, 0.984–0.996; *p* = .002), but no causal relationship was observed between PD and MDD (IVW method, OR = 0.974; 95% CI, 0.942–1.009; *p* = .141). In individuals of East Asian ancestry, no causal relationship was observed between PD and ever depressed for a whole week (IVW method, OR = 1.001; 95% CI, 0.829–1.209; *p* = .990) and between PD and MDD (IVW method, OR = 1.017; 95% CI, 0.982–1.052; *p* = .342). The results of the three additional analysis methods were similar to those of the IVW method, and there was no heterogeneity according to Cochran's *Q*‐test. There was no evidence of pleiotropy based on MR‐Egger intercept test and MR‐PRESSO. The FinnGen validation dataset supported these findings. The results are stable and reliable.

**Conclusion:**

The observed increase in depression among PD patients could potentially be attributed to modifiable acquired factors. Consequently, there is an urgent need to strengthen the management of PD patients in order to prevent the development of depression in the future.

## Graphical abstract

The present study aimed to employ Mendelian randomization (MR) to investigate the causal relationship between Parkinson's disease (PD) and the risk of depression severity across different populations. Our findings indicate that the observed increased depression in PD patients is likely attributable to modifiable acquired factors rather than being genetically determined.

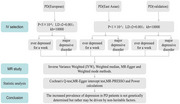

## 1 INTRODUCTION

Parkinson's disease (PD) is a common progressive neurodegenerative disorder primarily characterized by clinical symptoms such as tremors, rigidity, bradykinesia, and postural balance impairments (Bloem et al., 2021). With an aging population, its incidence is increasing yearly. The main pathological changes involve the progressive degeneration and loss of dopaminergic neurons in the substantia nigra pars compacta of the midbrain, along with the formation of Lewy bodies (Bloem et al., 2021). PD is also often accompanied by non‐motor symptoms such as autonomic dysfunction and neurocognitive impairments. Among these, depression is widely recognized as a common non‐motor symptom of PD (Cummings, 1992; Pontone & Mills, 2021; Zesiewicz & Hauser, 2002). Prolonged depression can also exacerbate PD's motor symptoms, posing a significant burden on families and society.

Depression is a relatively prevalent mental health disorder, primarily manifested by symptoms such as low mood, slowed thinking and speech, decreased attention and memory, and a loss of interest in nearly all activities. It has become a global health crisis that is growing in severity. The onset of depression is complex and related to both genetic and environmental factors, making it a stress‐related disease (Drevets et al., 2008). Studies have shown that many chronic illnesses are often comorbid with depression, not only in cardiovascular diseases like hypertension, coronary heart disease, and diabetes but also in neurodegenerative diseases like amyotrophic lateral sclerosis, Alzheimer's disease, Lewy body disease, and Huntington's disease (Zhang et al., 2018). However, it may be more prevalent and severe in PD (Ahmad et al., 2023; Hurley & Tizabi, 2013).

The prevalence of depression in PD is higher than in other chronic disabling diseases. Studies have shown that the prevalence of depression in PD patients is not only higher than in healthy individuals but also in diabetes patients (Gou et al., 2018; Tandberg et al., 1996). Moreover, the prevalence of depression in PD increases with disease duration (Weintraub et al., 2020). Across different studies, the reported prevalence of depression in PD varies widely, ranging from 2.7% to 90%, depending on diagnostic criteria, patient characteristics, and disease stage (Reijnders et al., 2008; Timmer et al., 2017). In studies using semi‐structured interviews to establish Diagnostic and Statistical Manual (DSM) criteria from the American Psychiatric Association, the reported prevalence of major depressive disorder (MDD) was 19%, while studies not using structured interviews for DSM criteria reported an MDD prevalence of 7%. However, clinically significant depressive symptoms were present in 35% of patients, regardless of whether they met DSM criteria for depressive disorder (Reijnders et al., 2008). Additionally, several meta‐analyses have reported a prevalence of depression in PD patients ranging from 30% to 50%, with an MDD prevalence between 14.0% and 22.9% (Becker et al., 2011; Chendo et al., 2022; Cong et al., 2022; Goodarzi et al., 2016). A review by Slaughter JR and other scholars also reported average prevalence rates of mood disorders, mild depression, and MDD at 22.5%, 36.6%, and 24.8%, respectively. These findings suggest that broader definitions of depression yield higher prevalence rates than MDD (Slaughter et al., 2001). Symptomatically, depression in PD differs from depression in the general population, with PD patients exhibiting more “discontent” and “loss of appetite” but fewer symptoms of “guilt”, “self‐hate”, and “loss of libido” (Kritzinger et al., 2015). Some studies have also found lower suicidal ideation in PD patients with depression compared to depressed patients without PD (Nazem et al., 2008).

However, it remains unclear whether this association is due to genetic or environmental factors or whether it results from confounding or shared risk factors such as aging, medication use, or long‐term psychological stress. Depression has a significant impact on the quality of life for both PD patients and caregivers, and it can lead to disability in PD patients (Ahmad et al., 2023). Early treatment of depression can slow disease progression and improve quality of life in PD patients, making it crucial for achieving positive treatment outcomes. However, diagnosing depression in PD is particularly challenging due to the high overlap between PD symptoms and depression symptoms. Therefore, understanding the potential relationship between PD and depression, improving diagnosis rates, and providing early treatment are crucial. However, observational studies alone struggle to establish the genetic association between PD, depression, and depression severity. Mendelian randomization (MR), a natural randomized controlled trial that estimates the causal effect between a specific exposure and outcome using genetic variants as instrumental variables (IVs), can be utilized to analyze the causal relationship between Parkinson's disease and depression severity (Davey Smith & Hemani, 2014). By using MR, we can minimize confounding factors like aging, medication exposure, or long‐term stress exposure.

In this study, we applied two‐sample MR using large‐scale genome‐wide association study (GWAS) summary statistics for PD, ever depressed for a whole week, and MDD. We not only stratified depression based on severity into ever depressed for a whole week and MDD but also categorized populations into European and East Asian ancestries to comprehensively elucidate the causal effect of PD on depression risk.

## 2 MATERIALS AND METHODS

This study involves a re‐analysis of previously collected and publicly available data; thus, no additional ethical approval is required.

### 2.1 GWAS data source

The GWAS summary data for PD in individuals of European ancestry were obtained from the International Parkinson's Disease Genomics Consortium, representing the largest sample size to date. The included population comprises 14 study cohorts, with 33,674 PD cases and 449,056 controls. For validation, we selected PD data from the latest version of the FinnGen dataset R9, which include 4235 PD cases and 373,042 controls (Table 1).

**TABLE 1:** Details of the genome‐wide association study (GWAS) included in Mendelian randomization.

Bloodline	Phenotype	Years	SNP	Source
**European ancestry**	PD (Nalls et al., 2019)	2019	17,891,936	https://gwas.mrcieu.ac.uk/
	PD verify	2023	20,170,236	https://www.FinnGen.fi/en/access_results
	Ever depressed for a whole week	2018	9,851,867	https://gwas.mrcieu.ac.uk/
	MDD (Wray et al., 2018)	2018	13,554,550	https://pgc.unc.edu/
**East Asian ancestry**	PD (Sakaue et al., 2021)	2021	13,435,531	https://pheweb.jp/
	Ever depressed for a whole week	2020	8,227,987	https://gwas.mrcieu.ac.uk/
	MDD (Giannakopoulou et al., 2021)	2021	7,922,500	https://pgc.unc.edu/

The GWAS summary data for PD in individuals of East Asian ancestry were obtained from the BioBank Japan PheWeb (BBJ), including 340 PD cases and 175,788 controls (Table 1).

To avoid the confounding effects of depression severity on the analysis, we selected two phenotypes in individuals of European ancestry: those who have ever depressed for a whole week and MDD. The GWAS summary data for the ever depressed for a whole week phenotype were obtained from the online database at the IEU Open GWAS Project, including 80,611 cases and 69,288 controls. The GWAS summary data for MDD, representing the largest sample size to date and more representative of MDD, were obtained from the Psychiatric Genomics Consortium database. Following the meta‐analysis by Howard et al. (2019), which included the “broad depression phenotype” (self‐reported seeking help for mental health difficulties) accounting for over 70% of cases, we chose to analyze the summary data from Wray et al. (2018) for a more accurate representation of MDD. Due to data restrictions from 23andMe, we selected 59,851 MDD cases and 113,154 controls (Table 1).

For East Asian ancestry individuals, the GWAS summary data for the ever depressed for a whole week phenotype were obtained from the online database at the IEU Open GWAS Project, including 458 cases and 661 controls. The GWAS summary data for MDD were obtained from the Psychiatric Genomics Consortium database, including 15,771 cases and 178,777 controls (Table 1).

### 2.2 Choice of instrumental variable (IV)

Single Nucleotide Polymorphisms（SNPs） should meet the following three assumptions: (1) Genetic variants should be strongly associated with the exposure, and the selected IVs should demonstrate a robust association with PD. (2) The genetic variants extracted for exposure should be independent of any confounding factors. (3) Genetic variants should influence outcomes only through the exposure. To satisfy these assumptions, we filtered SNPs through a rigorous process (Haycock et al., 2016).

Using the TwoSampleMR package (version 0.5.7), we identified SNPs associated with PD in individuals of European ancestry, employing a *p* value threshold of 5 × 10^−8^, and removed SNPs in linkage disequilibrium (LD *r* ^2^ = 0.001 and kb = 10,000). From these results, we extracted SNPs, sought proxy SNPs, and eliminated SNPs with palindromic structures. Subsequently, 22 and 17 IVs were selected for two‐sample MR to estimate the impact of PD on ever depressed for a whole week and MDD, respectively (Table S1–S2). When validating with PD in FinnGen datasets, using a *p* value threshold of 1 × 10^−5^, we identified 35 and 33 IVs for ever depressed for a whole week and MDD, respectively, and employed in MR analysis (Table S3–S4). For East Asian ancestry, after identifying SNPs associated with PD (using a *p* value threshold of 1 × 10^−5^) and following the aforementioned steps, six and five IVs were selected for two‐sample MR to estimate the influence of PD on ever depressed for a whole week and MDD in East Asian populations (Table S5–S6).

We calculated the *F*‐statistic for each IV to ascertain its strength, discarding weak IVs with an *F*‐statistic < 10 (Burgess & Thompson, 2011; Papadimitriou et al., 2020) (Table S1–S6).

### 2.3 Statistical analysis

Using R version 4.3.1 and TwoSampleMR (version 0.5.7), we combined effect estimates from individual SNPs using the inverse variance weighted (IVW) random‐effects model as the primary analysis. Additionally, we employed MR Egger, weighted median, and weighted mode to complement the findings, examining violations of MR assumptions. These diverse methods are utilized because they rest on different fundamental assumptions regarding horizontal pleiotropy.

Sensitivity analysis is paramount in MR studies for detecting pleiotropy. We assessed heterogeneity among SNPs using the Cochran's *Q*‐test, with *p *< .05 indicating heterogeneity. We evaluated pleiotropy among SNPs using the MR‐Egger intercept, where *p *< .05 suggests pleiotropy. Furthermore, we employed the outlier method (MR‐PRESSO) to assess horizontal pleiotropy. If horizontal pleiotropy is detected, we conduct further analysis using MR‐PRESSO with outlier correction (Verbanck et al., 2018). We then performed leave‐one‐out analysis to assess the impact of individual SNPs on the final results in IVW analysis. Finally, funnel plot analysis was conducted to observe symmetry in the funnel plot. Additionally, statistical results with significant causal relationships underwent POWER calculations on https://shiny.cnsgenomics.com/mRnd/, as statistical POWER indicates the likelihood of obtaining significant results (Brion et al., 2013).

## 3 RESULTS

### 3.1 The causal relationship between PD and ever depressed for a whole week in European ancestry

**FIGURE 1:**
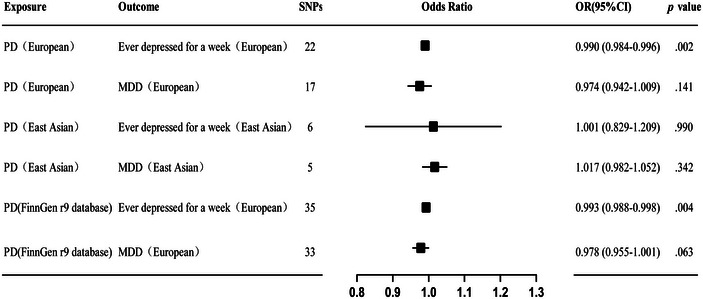
Forest plots showed the causal associations between depression severity and Parkinson's disease (PD) in different populations by inverse variance weighted (IVW). MDD, major depressive disorder; OR, odds ratio

**FIGURE 2:**
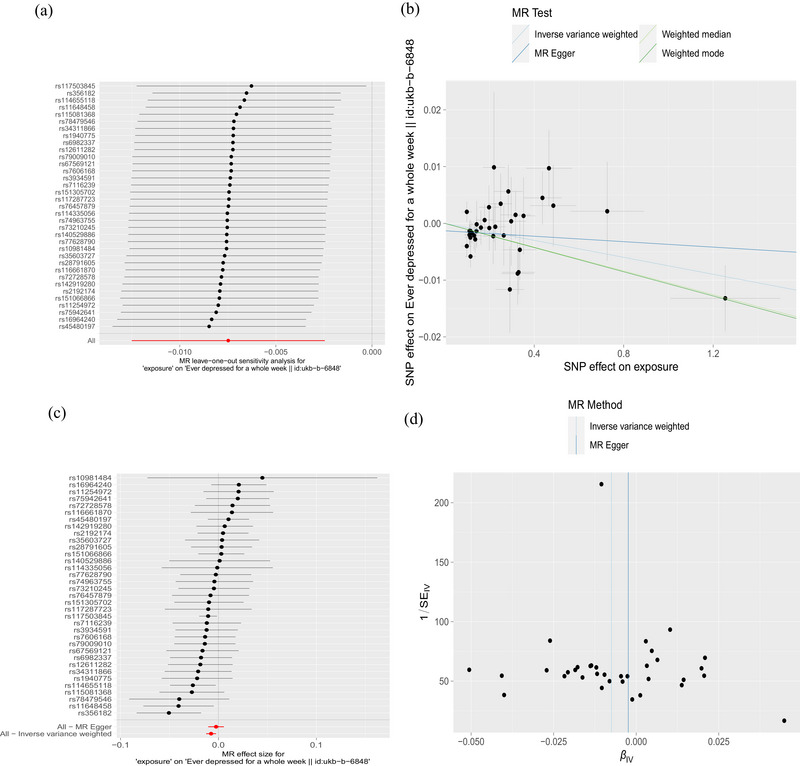
The causal relationship between Parkinson's disease (PD) and ever depressed for a whole week in European ancestry. (a) Mendelian randomization (MR) leave‐one‐out sensitivity analysis of PD's causal effect on having experienced depression for a whole week. The figure shows that excluding a particular SNP does not lead to a significant change in the overall result. (b) Scatter plot: The slope of the line corresponds to the causal estimate from each method. (c) The forest plot shows the estimate of the effect of genetically increased PD risk on ever depressed for a whole week risk. Each black dot represents the log odds ratio (OR) for ever depressed for a whole week per standard deviation (SD) increase in log OR for PD. Horizontal lines represent 95% confidence intervals (95% CIs). (d) Funnel plot showing the relationship between the causal effect of PD on ever depressed for a whole week estimated using each individual SNP as a separate instrument against the inverse of the standard error of the causal estimate. There is relatively symmetry in the plot.

Twenty‐two SNPs related to PD were used to investigate the causal effect of PD on risk of ever depressed for a whole week in European ancestry, and our findings suggest that PD may confer a protective effect against the risk of ever depressed for a whole week (IVW method, odds ratio [OR] = 0.990; 95% confidence interval [95% CI], 0.984–0.996; *p* = .002) (Figure 1). This result was further validated by MR‐Egger, weighted median, and weighted mode. Cochran's *Q*‐test for MR‐Egger had a *p* value of .279, and IVW had a *p* value of .267, indicating no heterogeneity. The MR‐Egger intercept test showed a *p* value of .294, indicating no pleiotropy. Additionally, MR‐PRESSO analysis showed a global *p* value of .237, indicating no outliers and no pleiotropy (Table 2). Furthermore, there was no strong violation of the overall effect of PD on depression symptoms by any individual SNP in the leave‐one‐out analysis. The funnel plot was also symmetric, indicating no pleiotropy (Figure 2). The validation analysis also produced consistent results, and suggested that PD may reduce the risk of having been depressed for a whole week (IVW method, OR = 0.993; 95% CI, 0.988–0.998; *p* = .004) (Figure 1), with no heterogeneity or pleiotropy detected (Table 3) (Figure S1).

**TABLE 2:** Two‐sample Mendelian randomization (MR) estimates for the effect of Parkinson's disease (PD) on ever depressed for a whole week and major depressive disorder (MDD).

Bloodline	Outcomes	nSNPs	Methods	OR (95%CI)	*p* value	MR‐PRESSO	Heterogeneity	Pleiotropy
European ancestry	Ever depressed for a week	22	MR Egger	0.982 (0.967–0.998)	.037	0.237	0.279	0.294
		22	Weighted median	0.989 (0.981–0.998)	.014			
		22	IVW	0.990 (0.984–0.996)	.002		0.267	
		22	Weighted mode	0.982 (0.967–0.997)	.031			
	MDD	17	MR Egger	0.951 (0.875–1.034)	.254	0.072	0.128	0.533
		17	Weighted median	0.979 (0.935–1.026)	.371			
		17	IVW	0.974 (0.942–1.009)	.141		0.148	
		17	Weighted mode	0.972 (0.911–1.037)	.405			
East Asian ancestry	Ever depressed for a week	6	MR Egger	0.994 (0.652–1.517)	.980	0.889	0.708	0.972
		6	Weighted median	0.992 (0.774–1.270)	.947			
		6	IVW	1.001 (0.829–1.209)	.990		0.828	
		6	Weighted mode	0.983 (0.741–1.305)	.913			
	MDD	5	MR Egger	1.067 (0.988–1.153)	.198	0.55	0.645	0.265
		5	Weighted median	1.032 (0.989–1.077)	.143			
		5	IVW	1.017 (0.982–1.052)	.342		0.473	
		5	Weighted mode	1.036 (0.981–1.094)	.270			

*Note*: Heterogeneity, *p* value for Cochran's *Q*‐test; Pleiotropy, *p* value for MR‐Egger intercept test; MR‐PRESSO, MR‐PRESSO global *p* value.

Abbreviations: IVW, inverse variance weighted; OR, odds ratio.

**TABLE 3:** Two‐sample Mendelian randomization (MR) estimates for the effect of Parkinson's disease (PD) on ever depressed for a whole week and major depressive disorder (MDD) (PD from FinnGen r9 database).

Outcomes	nSNPs	Methods	OR (95% CI)	*p* value	MR‐PRESSO	Heterogeneity	Pleiotropy
Ever depressed for a week	35	MR Egger	0.998 (0.990–1.006)	.559	0.231	0.419	0.121
	35	Weighted median	0.990 (0.982–0.998)	.012			
	35	IVW	0.993 (0.988–0.998)	.004		0.348	
	35	Weighted mode	0.989 (0.980–0.999)	.032			
**MDD**	33	MR Egger	0.986 (0.949–1.025)	.483	0.432	0.326	0.597
	33	Weighted median	0.987 (0.951–1.025)	.500			
	33	IVW	0.978 (0.955–1.001)	.063		0.359	
	33	Weighted mode	0.995 (0.958–1.033)	.785			

*Note*: Heterogeneity, *p* value for Cochran's *Q*‐test; Pleiotropy, *p* value for MR‐Egger intercept test; MR‐PRESSO, MR‐PRESSO global *p* value.

Abbreviations: IVW, inverse variance weighted; OR, odds ratio.

### 3.2 The causal relationship between PD and MDD in European ancestry

**FIGURE 3:**
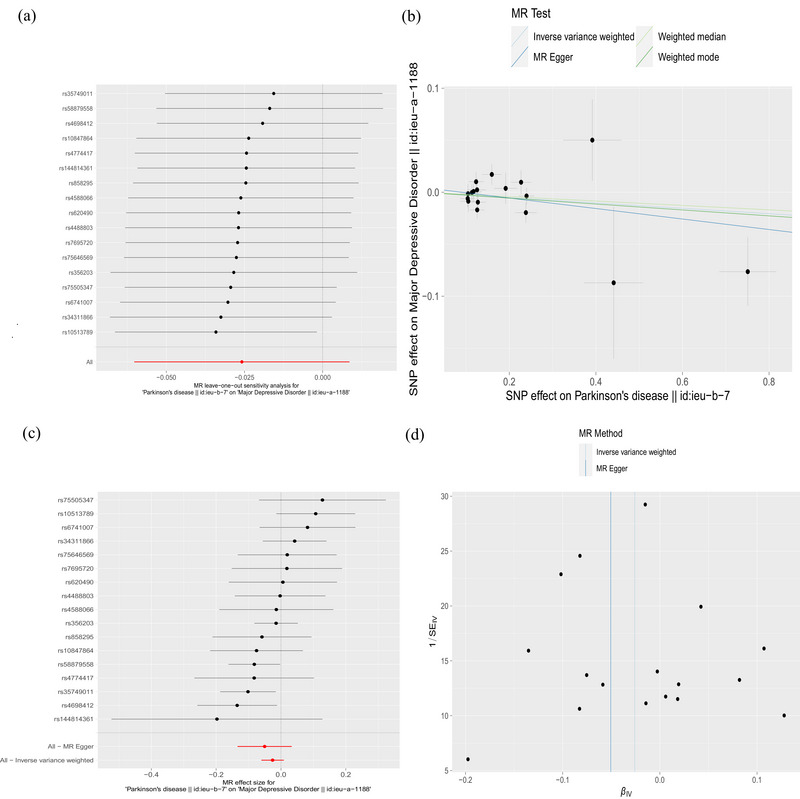
The causal relationship between Parkinson's disease (PD) and major depressive disorder (MDD) in European ancestry. (a) Mendelian randomization (MR) leave‐one‐out sensitivity analysis for PD on MDD. The figure shows that excluding a particular SNP does not lead to a significant change in the overall result. (b) Scatter plot: The slope of the line corresponds to the causal estimate from each method. (c) The forest plot shows the estimate of the effect of genetically increased PD risk on MDD risk. Each black dot represents the log odds ratio (OR) for MDD per standard deviation (SD) increase in log OR for PD. Horizontal lines represent 95% confidence intervals (95% CIs). (d) Funnel plot showing the relationship between the causal effect of PD on MDD estimated using each individual SNP as a separate instrument against the inverse of the standard error of the causal estimate. There is relatively symmetry in the plot.

Seventeen SNPs related to PD were used to investigate the causal effect of PD on risk of MDD in European ancestry, and no significant causal relationship was observed (IVW method, OR = 0.974; 95% CI, 0.942–1.009; *p* = .141) (Figure 1). This result was further validated by MR‐Egger, weighted median, and weighted mode. Cochran's *Q*‐test for MR‐Egger had a *p* value of .128, and IVW had a *p* value of .148, indicating no heterogeneity. The MR‐Egger intercept test showed a *p* value of .533, indicating no pleiotropy. Additionally, MR‐PRESSO analysis showed a global *p* value of .;072, indicating no outliers and no pleiotropy (Table 2). Furthermore, there was no strong violation of the overall effect of PD on depression symptoms by any individual SNP in the leave‐one‐out analysis. The funnel plot was also symmetric, indicating no pleiotropy (Figure 3).The validation analysis also produced consistent results (Figure 1), indicating no causal relationship between PD and risk of MDD with no heterogeneity or pleiotropy detected (Table 3) (Figure S2).

### 3.3 The causal relationship between PD and ever depressed for a whole week in East Asian ancestry

**FIGURE 4:**
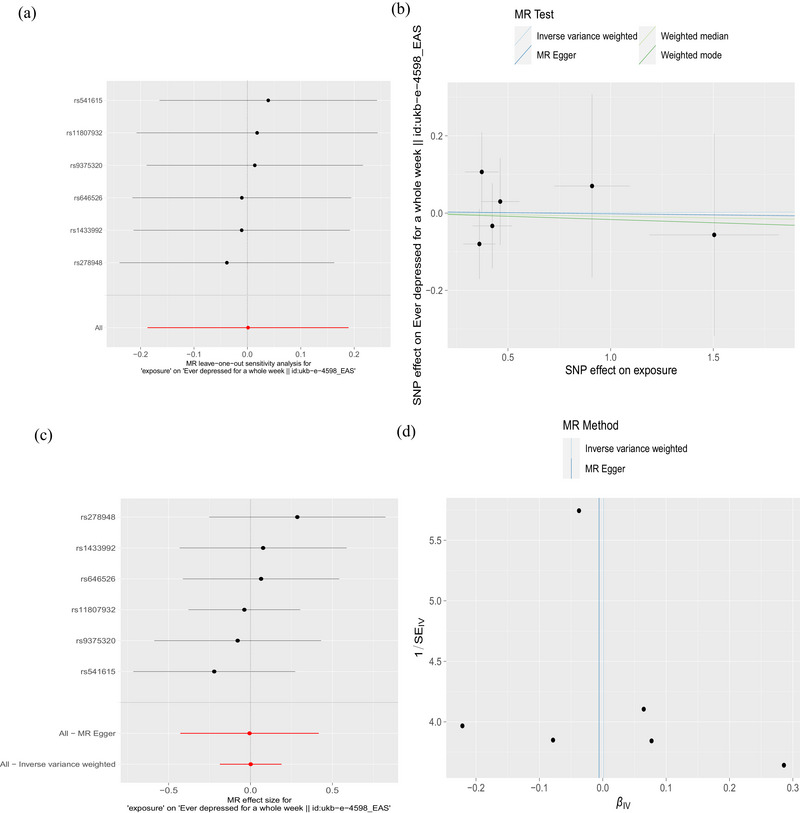
The causal relationship between Parkinson's disease (PD) and ever depressed for a whole week in East Asian ancestry. (a) Mendelian randomization (MR) leave‐one‐out sensitivity analysis of PD's causal effect on having experienced depression for a whole week. The figure shows that excluding a particular SNP does not lead to a significant change in the overall result. (b) Scatter plot: The slope of the line corresponds to the causal estimate from each method. (c) The forest plot shows the estimate of the effect of genetically increased PD risk on ever depressed for a whole week risk. Each black dot represents the log odds ratio (OR) for ever depressed for a whole week per standard deviation (SD) increase in log OR for PD. Horizontal lines represent 95% confidence intervals (95% CIs). (d) Funnel plot showing the relationship between the causal effect of PD on ever depressed for a whole week estimated using each individual SNP as a separate instrument against the inverse of the standard error of the causal estimate. There is relatively symmetry in the plot.

Six SNPs related to PD were used to investigate the causal effect of PD on risk of ever depressed for a whole week in East Asian ancestry, and no significant causal relationship was observed (IVW method, OR = 1.001; 95% CI, 0.829–1.209; *p* = .990) (Figure 1). This result was further validated by MR‐Egger, weighted median, and weighted mode. Cochran's *Q*‐test for MR‐Egger had a *p* value of .708, and IVW had a *p* value of .828, indicating no heterogeneity. The MR‐Egger intercept test showed a *p* value of .972, indicating no pleiotropy. Additionally, MR‐PRESSO analysis showed a global *p* value of .55, indicating no outliers and no pleiotropy (Table 2). Furthermore, there was no strong violation of the overall effect of PD on depression symptoms by any individual SNP in the leave‐one‐out analysis. The funnel plot was also symmetric, indicating no pleiotropy (Figure 4).

### 3.4 The causal relationship between PD and MDD in East Asian ancestry

**FIGURE 5:**
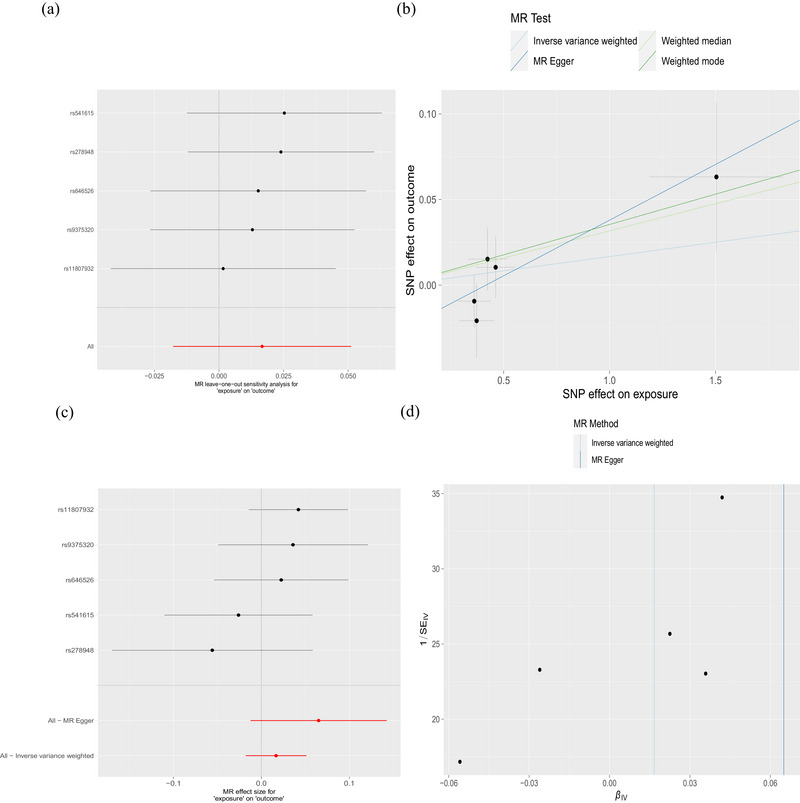
The causal relationship between Parkinson's disease (PD) and major depressive disorder (MDD) in East Asian ancestry. (a) Mendelian randomization (MR) leave‐one‐out sensitivity analysis for PD on MDD. The figure shows that excluding a particular SNP does not lead to a significant change in the overall result. (b) Scatter plot: The slope of the line corresponds to the causal estimate from each method. (c) The forest plot shows the estimate of the effect of genetically increased PD risk on MDD risk. Each black dot represents the log odds ratio (OR) for MDD per standard deviation (SD) increase in log OR for PD. Horizontal lines represent 95% confidence intervals (95% CIs). (d) Funnel plot showing the relationship between the causal effect of PD on MDD estimated using each individual SNP as a separate instrument against the inverse of the standard error of the causal estimate. There is relatively symmetry in the plot.

Five SNPs related to PD were used to investigate the causal effect of PD on risk of MDD in East Asian ancestry, and no significant causal relationship was observed (OR = 1.017; 95% CI, 0.982–1.052; *p* = .342) (Figure 1). The same results were obtained from further MR‐Egger, weighted median, and weighted mode. Cochran's *Q*‐test for MR‐Egger gave a *p* value of .645, and the IVW *p* value was .473, indicating no heterogeneity. The MR‐Egger intercept test showed a *p* value of .265, indicating no pleiotropy. Additionally, MR‐PRESSO analysis showed a global *p* value of .889, indicating no outliers and no pleiotropy (Table 2). Furthermore, there was no single SNP that strongly violated the overall effect of PD on depressive symptoms in the leave‐one‐out analysis. The funnel plot was symmetric and showed no pleiotropy (Figure 5).

## 4 DISCUSSION

This study provides the first evidence that, in individuals of European ancestry, genetically predicted PD may confer a protective effect against the risk of ever depressed for a whole week, with a causal relationship identified (IVW method, OR = 0.990; 95% CI, 0.984–0.996; *p* = .002). Similar findings were observed in validation analyses (IVW method, OR = 0.993; 95% CI, 0.988–0.998; *p* = .004). However, no causal relationship was identified between PD and the risk of MDD. In individuals of East Asian ancestry, no causal relationship was observed between PD and the risk of ever depressed for a whole week or MDD, with consistent results in validation analyses. All analyses showed no heterogeneity or pleiotropy, indicating stable and reliable results.

The pathogenesis of depression in PD remains incompletely understood, with potential contributions from psychosocial factors, changes in brain structure and function, and genetic factors. Some studies suggest that depression in PD may be more likely due to neuropathological changes in the brain rather than purely psychological factors (Menza et al., 1999). Compared to non‐depressed PD patients, depressed PD patients exhibit increased cortical area in the orbitofrontal and insular regions, which also corresponds to white matter atrophy and alterations in integrity and functional connectivity in these regions (Huang et al., 2016). Other scholars propose that depression may be related to the neurodegenerative process in PD, where varying degrees of degeneration of subcortical neurons, such as dopamine, serotonin, and norepinephrine, contribute to the development of depression (McDonald et al., 2003). Studies have also confirmed that the occurrence of depression in PD patients is associated with specific losses of dopamine and norepinephrine innervation in both cortical and subcortical components of the limbic system (Remy et al., 2005). Postmortem examinations of PD patients with depression have revealed reductions in serotonin neurons in the dorsal raphe nucleus and dopamine neurons in the ventral tegmental area (Brown & Gershon, 1993; Paulus & Jellinger, 1991). Additionally, research indicates that the occurrence of PD depression is closely related to neurotransmitter imbalances (Ahmad et al., 2023). Numerous studies have identified an association between polymorphisms in the serotonin transporter gene promoter region (5HTTLPR) and the development of depression, with the short allele and S/S genotype of 5HTTLPR potentially serving as risk factors for depression in PD patients (Cheng et al., 2021; Menza et al., 1999; Mössner et al., 2001; Wang et al., 2019). A recent meta‐analysis also indicates that variations in glucosidase beta acid（GBA） are associated with depressive symptoms in PD patients (D'Souza & Rajkumar, 2020). Furthermore, research suggests that genetic polymorphisms in the circadian rhythm gene thyrotrophin embryonic factor rs738499 are related to depressive symptoms in PD (Hua et al., 2012). PD patients carrying LRRK2 mutations are more susceptible to depression (Marras et al., 2011). However, some studies suggest that the occurrence of depression may be independent of genetic makeup (Burn et al., 2006; Dissanayaka et al., 2009).

The findings from this study, where genetically predicted PD in individuals of European ancestry may confer a protective effect against the risk of ever depressed for a whole week but with low statistical power (POWER calculated value of 0.05), and the absence of a causal relationship between PD and MDD risk as well as between PD and depression in East Asian ancestry, lead us to consider that the increased incidence of depression in PD patients may be more attributable to modifiable environmental factors. These factors may include reactions to PD‐related disability symptoms, psychological stress factors, poor quality of life or social factors, or age factors rather than genetic factors. Mounting evidence increasingly suggests that psychological stress factors not only represent a risk factor for depression but may also play a role in the pathogenesis of Parkinson's disease (van Wamelen et al., 2020). Studies have shown that functional decline, rapid disease progression, and long‐term use of levodopa are also associated with depressive symptoms in PD patients (Custodio et al., 2018), with the incidence of depression increasing with age. A recent meta‐analysis indicates that factors such as average or poor self‐perceived health status, dysfunction, and negative life events appear to be important risk factors for depression in older adults (Qiu et al., 2020). Therefore, we may be able to implement interventions focused on modifiable factors to reduce the incidence of depression in PD patients, such as enhancing mental health education for PD patients and improving their quality of life.

In recent years, previous scholars analyzing the causal association between PD and MDD have, similarly to our findings, not identified any causal relationship. Kim et al. (2021) also mentioned in their analysis of PD and schizophrenia that the association between PD and the other seven mental illnesses, including MDD, was not significant. However, the MDD GWAS data they used were from 2013, with a small sample size, and lacked detailed analysis. Wu et al. (2023) analyzed the relationship between mental illness and PD in European populations, conducting reverse research, and found no causal relationship in MDD analysis results. Additionally, the MDD GWAS they chose was from the meta‐analysis of Howard et al. (2019), which included the “broad depression phenotype” (self‐reported seeking help due to mental health difficulties) accounting for over 70% of the cases, and thus could not represent the phenotype of “MDD” well.

This study is the first to stratify depression severity into ever depressed for a whole week and MDD, and to conduct a comprehensive MR analysis of PD and depression for different ethnic groups, namely European and East Asian populations. The GWAS sample size selected in this study is large, and validation analysis has been conducted to analyze the relationships between PD and ever depressed for a whole week and MDD, respectively, avoiding the impact of depression severity. Additionally, analysis of East Asian populations has been conducted to avoid differences in results caused by different populations, making our results applicable to both European and East Asian populations. However, there are some limitations to this study. First, in our analysis of PD with ever depressed for a whole week and MDD in European populations, we selected almost exclusively SNPs with genome‐wide significance levels (*p *< 5 × 10^−8^), ignoring true‐related variants that did not meet the strict p value threshold. However, we used a more permissive *p *< 1 × 10^−5^ in our validation analysis. Second, for the analysis of East Asian populations, the sample size was smaller, with fewer IVs available for analysis, and no validation analysis was conducted. Therefore, larger samples are needed to validate our conclusions in the future.

Although our results contradict previous observational studies, we cannot deny the causal relationship between PD and depression. The relationship between PD and depression remains highly complex and requires further analysis with larger sample sizes and stricter disease diagnoses.

## 5 CONCLUSION

Upon conducting an extensive MR analysis, we observed that in European populations, genetically predicted PD exerts a slight protective effect against ever depressed for a whole week. Surprisingly, there is no causal relationship between PD and MDD in European populations, as well as between PD and ever depressed for a whole week or MDD in East Asian populations. These findings suggest that the heightened susceptibility to depression among PD patients is not genetically determined but rather may be driven by non‐heritable factors. Such factors may encompass responses to disabling PD symptoms, psychological stressors, compromised quality of life, or societal influences. Given these insights, efforts to mitigate depression incidence in PD patients could prioritize non‐genetic intervention strategies, including bolstering mental health education initiatives tailored for this population and enhancing overall quality of life. Future investigations into depression comorbidity in PD are likely to shift their emphasis toward identifiable and modifiable risk factors.

## AUTHOR CONTRIBUTIONS

**Yidan Qin**: Conceptualization; methodology; software; validation; formal analysis; writing—original draft; writing—review and editing; investigation. **Jia Li**: Conceptualization; methodology; investigation; data curation; writing—review and editing. **Wei Quan**: Validation; resources; data curation; writing—review and editing. **Jia Song**: Writing—review and editing; formal analysis; validation. **Jing Xu**: Conceptualization; writing—review and editing; visualization. **Jiajun Chen**: Conceptualization; writing—review and editing; funding acquisition; supervision.

## CONFLICT OF INTEREST STATEMENT

The authors declare no conflicts of interest.

### PEER REVIEW

The peer review history for this article is available at https://publons.com/publon/10.1002/brb3.3642.

## Supporting information


**Supplementary Table 1**. Summarized data of SNPs finally identified as IVs in our MR analyses (The causal relationship between PD and ever depressed for a whole week in European ancestry)
**Supplementary Table 2**. Summarized data of SNPs finally identified as IVs in our MR analyses (The causal relationship between PD and MDD in European ancestry)
**Supplementary Table 3**. Summarized data of SNPs finally identified as IVs in our MR analyses (The causal relationship between PD and ever depressed for a whole week. PD GWAS data comes from FinnGen datasets for validation analysis)
**Supplementary Table 4**. Summarized data of SNPs finally identified as IVs in our MR analyses (The causal relationship between PD and MDD.PD GWAS data comes from FinnGen datasets for validation analysis)
**Supplementary Table 5**. Summarized data of SNPs finally identified as IVs in our MR analyses (The causal relationship between PD and ever depressed for a whole week in East Asian ancestry)
**Supplementary Table 6**. Summarized data of SNPs finally identified as IVs in our MR analyses (The causal relationship between PD and MDD in East Asian ancestry)
**Supplementary Figure 1**. The causal relationship between PD and ever depressed for a whole week (PD from FinnGen r9 database). Supplementary Figure2. The causal relationship between PD and MDD(PD from FinnGen r9 database)

## Data Availability

The GWAS data used during the current study can be obtained from cited study authors on reasonable request. European ancestry: the data on PD can be found at this link: https://gwas.mrcieu.ac.uk/; https://www.FinnGen.fi/en/access_results (confirmation analysis). The data on ever depressed for a whole week can be found at: https://gwas.mrcieu.ac.uk/. The data on MDD can be found at: https://gwas.mrcieu.ac.uk/. East Asian ancestry: the data on PD can be found at: https://pheweb.jp/. The data on ever depressed for a whole week can be found at: https://gwas.mrcieu.ac.uk/. The data on MDD can be found at: https://pgc.unc.edu/.
