# Supplementary material for: Risk of Parkinson's disease and depression severity in different populations: A two‐sample Mendelian randomization analysis
Source: Brain Behav. 2024 Sep 1;14(9):e3642. doi: 10.1002/brb3.3642 (PMC11366827; doi:10.1002/brb3.3642)
Supplement: Supplementary file 1 — Supplementary Table 1. Summarized data of SNPs finally identified as IVs in our MR analyses (The causal relationship between PD and ever depressed for a whole week in European ancestry) Supplementary Table 2. Summarized data of SNPs finally identified as IVs in our MR analyses (The causal relationship between PD and MDD in European ancestry) Supplementary Table 3. Summarized data of SNPs finally identified as IVs in our MR analyses (The causal relationship between PD and ever depressed for a whole week. PD GWAS data comes from FinnGen datasets for validation analysis) Supplementary Table 4. Summarized data of SNPs finally identified as IVs in our MR analyses (The causal relationship between PD and MDD.PD GWAS data comes from FinnGen datasets for validation analysis) Supplementary Table 5. Summarized data of SNPs finally identified as IVs in our MR analyses (The causal relationship between PD and ever depressed for a whole week in East Asian ancestry) Supplementary Table 6. Summarized data of SNPs finally identified as IVs in our MR analyses (The causal relationship between PD and MDD in East Asian ancestry) Supplementary Figure 1. The causal relationship between PD and ever depressed for a whole week (PD from FinnGen r9 database). Supplementary Figure2. The causal relationship between PD and MDD(PD from FinnGen r9 database) [file BRB3-14-e3642-s001.pdf]

Supplementary Material

1 Supplementary Figures and Tables

1.1 Supplementary Tables

Supplementary Table 1. Summarized data of SNPs finally identified as IVs in our MR analyses（The causal relationship between PD and ever depressed for a whole week in European ancestry）

exposure	chr	pos	beta	se	*p*val	SNP	effect_allele	other_allele	eaf	r2	F
PD	1	155135036	0.7508	0.0659	5.02E-30	rs35749011	A	G	0.0191	0.000268817	129.8003607
PD	1	205656453	-0.1492	0.0239	4.10E-10	rs823106	C	G	0.8488	8.07E-05	38.97086496
PD	2	169119609	0.1784	0.0248	6.21E-13	rs4613239	G	C	0.1326	0.000107185	51.746924
PD	2	135537119	-0.1233	0.0175	2.09E-12	rs6741007	G	T	0.4507	0.000102826	49.64188412
PD	3	58218352	-0.1136	0.0199	1.08E-08	rs4488803	A	G	0.3746	6.75E-05	32.5873249
PD	3	182760073	-0.1596	0.0219	3.18E-13	rs10513789	G	T	0.1826	0.000110008	53.10993196
PD	4	77183300	-0.1255	0.0208	1.53E-09	rs7695720	C	A	0.2091	7.54E-05	36.40482791
PD	4	951947	0.2272	0.0231	7.97E-23	rs34311866	C	T	0.1958	0.000200356	96.73661688
PD	4	15737348	0.1258	0.0168	7.05E-14	rs4698412	A	G	0.553	0.000116142	56.07133798
PD	4	90666041	-0.2398	0.0178	3.01E-41	rs356203	T	C	0.6169	0.000375829	181.4916101
PD	5	60345424	0.1916	0.0266	5.62E-13	rs75646569	G	T	0.1117	0.000107467	51.88310236
PD	6	32561334	-0.2	0.0303	3.93E-11	rs35265698	G	C	0.1547	9.06E-05	43.56853199
PD	7	23245569	-0.1039	0.0176	3.83E-09	rs858295	G	A	0.3947	7.22E-05	34.85009451
PD	8	16697579	-0.1174	0.019	6.46E-10	rs620490	G	T	0.2762	7.91E-05	38.1792324

PD	10	121410917	0.4411	0.068	9.07E-11	rs144814361	T	C	0.0174	8.72E-05	42.07794202
PD	11	133764666	-0.1133	0.0178	1.94E-10	rs329647	C	G	0.6662	8.39E-05	40.51520267
PD	12	40885549	0.3917	0.0674	6.12E-09	rs75505347	T	C	0.0195	7.00E-05	33.77423732
PD	12	123326598	0.1274	0.0179	9.81E-13	rs10847864	T	G	0.3625	0.000104926	50.65601184
PD	15	61993702	0.1052	0.0192	4.63E-08	rs4774417	A	G	0.7397	6.22E-05	30.02114298
PD	16	30923602	0.1215	0.0184	4.33E-11	rs12934900	T	A	0.6571	9.03E-05	43.60287346
PD	17	44095467	-0.2383	0.025	1.36E-21	rs58879558	C	T	0.2229	0.000188184	90.85864756
PD	18	40672964	0.1046	0.0178	4.45E-09	rs4588066	A	G	0.326	7.15E-05	34.53198671

Supplementary Table 2. Summarized data of SNPs finally identified as IVs in our MR analyses（The causal relationship between PD and MDD in European ancestry）

exposure	chr	pos	beta	se	pval	SNP	effect_allele	other_allele	eaf	r2	F
PD	1	155135036	0.7508	0.0659	5.02E-30	rs35749011	A	G	0.0191	0.000268817	129.8003607
PD	2	135537119	-0.1233	0.0175	2.09E-12	rs6741007	G	T	0.4507	0.000102826	49.64188412
PD	3	58218352	-0.1136	0.0199	1.08E-08	rs4488803	A	G	0.3746	6.75E-05	32.5873249
PD	3	182760073	-0.1596	0.0219	3.18E-13	rs10513789	G	T	0.1826	0.000110008	53.10993196
PD	4	951947	0.2272	0.0231	7.97E-23	rs34311866	C	T	0.1958	0.000200356	96.73661688
PD	4	15737348	0.1258	0.0168	7.05E-14	rs4698412	A	G	0.553	0.000116142	56.07133798
PD	4	90666041	-0.2398	0.0178	3.01E-41	rs356203	T	C	0.6169	0.000375829	181.4916101
PD	5	60345424	0.1916	0.0266	5.62E-13	rs75646569	G	T	0.1117	0.000107467	51.88310236
PD	7	23245569	-0.1039	0.0176	3.83E-09	rs858295	G	A	0.3947	7.22E-05	34.85009451
PD	8	16697579	-0.1174	0.019	6.46E-10	rs620490	G	T	0.2762	7.91E-05	38.1792324
PD	10	121410917	0.4411	0.068	9.07E-11	rs144814361	T	C	0.0174	8.72E-05	42.07794202
PD	12	40885549	0.3917	0.0674	6.12E-09	rs75505347	T	C	0.0195	7.00E-05	33.77423732
PD	12	123326598	0.1274	0.0179	9.81E-13	rs10847864	T	G	0.3625	0.000104926	50.65601184
PD	15	61993702	0.1052	0.0192	4.63E-08	rs4774417	A	G	0.7397	6.22E-05	30.02114298
PD	17	44095467	-0.2383	0.025	1.36E-21	rs58879558	C	T	0.2229	0.000188184	90.85864756
PD	18	40672964	0.1046	0.0178	4.45E-09	rs4588066	A	G	0.326	7.15E-05	34.53198671

Supplementary Table 3. Summarized data of SNPs finally identified as IVs in our MR analyses（The causal relationship between PD and ever depressed for a whole week.PD GWAS data comes from FinnGen datasets for validation analysis）

chr	pos	other_allele	effect_allele	SNP	*p*val.	beta	se	eaf	r2	F
15	156038197	G	A	rs35603727	1.05E-11	0.352222	0.0518034	0.0387013	0.000122519	46.22896029
25	188522802	C	A	rs78479546	3.68E-06	0.29107	0.0628854	0.0267354	5.68E-05	21.42362383
30	46974783	C	T	rs116661870	1.17E-06	0.250119	0.0514629	0.0412752	6.26E-05	23.62123247
34	208686193	T	C	rs7606168	3.06E-06	0.12115	0.025962	0.749349	5.77E-05	21.7755054
39	73208801	G	A	rs75942641	8.34E-06	-0.284242	0.0637833	0.0364883	5.26E-05	19.85913045
42	958159	T	C	rs34311866	3.14E-07	0.136197	0.0266286	0.206332	6.93E-05	26.15990201
48	89704960	G	A	rs356182	5.93E-07	-0.115189	0.0230678	0.653231	6.61E-05	24.93488439
86	2669130	C	T	rs79009010	9.02E-06	0.335603	0.0755971	0.0188133	5.22E-05	19.70784305
87	24052835	A	C	rs67569121	9.70E-06	0.131212	0.0296603	0.153936	5.19E-05	19.57013085
94	171440573	A	G	rs3934591	5.67E-07	0.110766	0.0221435	0.509392	6.63E-05	25.02177474
98	1698687	T	A	rs142919280	2.35E-06	0.485462	0.102845	0.00876918	5.91E-05	22.28136609
105	17469090	A	G	rs115081368	8.04E-06	-0.325774	0.0729774	0.0288219	5.28E-05	19.92754348
110	32591414	A	G	rs114335056	8.58E-06	-0.142778	0.0320842	0.150869	5.25E-05	19.80331321
112	79692737	C	A	rs151305702	1.17E-06	-0.141871	0.0291887	0.185532	6.26E-05	23.62410976
114	83157474	G	A	rs45480197	2.84E-07	0.43677	0.0850829	0.0132396	6.98E-05	26.35231244
122	111751871	A	G	rs114655118	5.78E-06	-0.330154	0.0728121	0.0286802	5.45E-05	20.56002842
141	23836974	T	G	rs6982337	3.72E-07	0.112032	0.0220419	0.49328	6.85E-05	25.83353395
145	38433582	G	T	rs117287723	8.34E-06	0.216844	0.0486615	0.0479734	5.26E-05	19.8573611
152	136527647	T	C	rs72728578	8.41E-06	-0.197843	0.0444137	0.0778154	5.26E-05	19.84290102
156	112704677	C	T	rs10981484	9.39E-06	0.220072	0.0496684	0.0445246	5.20E-05	19.63210817
159	7054186	A	G	rs11254972	7.29E-06	0.0986691	0.0220002	0.492388	5.33E-05	20.1143857

292	1620419	C	T	rs7116239	2.71E-06	-0.1154	0.0245973	0.735072	5.83E-05	22.01072964
294	103814582	T	C	rs151066866	8.30E-06	0.72633	0.162955	0.00316289	5.27E-05	19.86690803
295	106116012	C	G	rs1940775	9.62E-06	-0.114542	0.0258819	0.77632	5.19E-05	19.58551122
316	21405286	A	G	rs2192174	2.57E-06	-0.316587	0.0673226	0.0329303	5.86E-05	22.11373457
347	101533791	T	C	rs76457879	2.51E-06	0.263835	0.0560467	0.0346616	5.87E-05	22.1596257
349	94471104	G	A	rs117503845	3.46E-07	-1.25392	0.246042	0.00478529	6.88E-05	25.97280527
351	389550	T	C	rs11648458	7.47E-06	0.0985747	0.022004	0.523282	5.32E-05	20.06898245
357	22255975	A	G	rs28791605	2.62E-06	0.178256	0.0379358	0.0842028	5.85E-05	22.07941271
3288	15845369	G	A	rs12611282	1.35E-06	0.11039	0.0228444	0.358963	6.19E-05	23.35057893
3292	30491631	A	C	rs16964240	5.27E-06	-0.465772	0.102284	0.0158528	5.50E-05	20.7361939
3296	5695328	A	C	rs74963755	2.48E-06	0.199245	0.0423017	0.066964	5.88E-05	22.18487159
3298	46046656	G	C	rs140529886	5.93E-06	0.296952	0.0655669	0.02359	5.44E-05	20.51166456
3301	18295032	T	A	rs73210245	1.77E-06	-0.161834	0.0338704	0.133971	6.05E-05	22.82950966
3305	27198147	T	G	rs77628790	8.17E-07	0.225982	0.0458256	0.0543639	6.45E-05	24.31806829

Supplementary Table 4. Summarized data of SNPs finally identified as IVs in our MR analyses（The causal relationship between PD and MDD.PD GWAS data comes from FinnGen datasets for validation analysis）

chr	pos	other_allele	effect_allele	SNP	*p*val	beta	se.	eaf	r2	F
15	156038197	G	A	rs35603727	1.05E-11	0.352222	0.0518034	0.0387013	0.000122519	46.22896029
25	188522802	C	A	rs78479546	3.68E-06	0.29107	0.0628854	0.0267354	5.68E-05	21.42362383
30	46974783	C	T	rs116661870	1.17E-06	0.250119	0.0514629	0.0412752	6.26E-05	23.62123247
34	208686193	T	C	rs7606168	3.06E-06	0.12115	0.025962	0.749349	5.77E-05	21.7755054
39	73208801	G	A	rs75942641	8.34E-06	-0.284242	0.0637833	0.0364883	5.26E-05	19.85913045
42	958159	T	C	rs34311866	3.14E-07	0.136197	0.0266286	0.206332	6.93E-05	26.15990201
48	89704960	G	A	rs356182	5.93E-07	-0.115189	0.0230678	0.653231	6.61E-05	24.93488439
86	2669130	C	T	rs79009010	9.02E-06	0.335603	0.0755971	0.0188133	5.22E-05	19.70784305
87	24052835	A	C	rs67569121	9.70E-06	0.131212	0.0296603	0.153936	5.19E-05	19.57013085
94	171440573	A	G	rs3934591	5.67E-07	0.110766	0.0221435	0.509392	6.63E-05	25.02177474
105	17469090	A	G	rs115081368	8.04E-06	-0.325774	0.0729774	0.0288219	5.28E-05	19.92754348
110	32591414	A	G	rs114335056	8.58E-06	-0.142778	0.0320842	0.150869	5.25E-05	19.80331321
112	79692737	C	A	rs151305702	1.17E-06	-0.141871	0.0291887	0.185532	6.26E-05	23.62410976
114	83157474	G	A	rs45480197	2.84E-07	0.43677	0.0850829	0.0132396	6.98E-05	26.35231244
122	111751871	A	G	rs114655118	5.78E-06	-0.330154	0.0728121	0.0286802	5.45E-05	20.56002842
141	23836974	T	G	rs6982337	3.72E-07	0.112032	0.0220419	0.49328	6.85E-05	25.83353395
145	38433582	G	T	rs117287723	8.34E-06	0.216844	0.0486615	0.0479734	5.26E-05	19.8573611
152	136527647	T	C	rs72728578	8.41E-06	-0.197843	0.0444137	0.0778154	5.26E-05	19.84290102
156	112704677	C	T	rs10981484	9.39E-06	0.220072	0.0496684	0.0445246	5.20E-05	19.63210817
159	7054186	A	G	rs11254972	7.29E-06	0.0986691	0.0220002	0.492388	5.33E-05	20.1143857
206	115531529	T	C	rs10444100	5.67E-06	0.104042	0.0229253	0.622226	5.46E-05	20.59610166

292	1620419	C	T	rs7116239	2.71E-06	-0.1154	0.0245973	0.735072	5.83E-05	22.01072964
294	103814582	T	C	rs151066866	8.30E-06	0.72633	0.162955	0.00316289	5.27E-05	19.86690803
316	21405286	A	G	rs2192174	2.57E-06	-0.316587	0.0673226	0.0329303	5.86E-05	22.11373457
347	101533791	T	C	rs76457879	2.51E-06	0.263835	0.0560467	0.0346616	5.87E-05	22.1596257
349	94471104	G	A	rs117503845	3.46E-07	-1.25392	0.246042	0.00478529	6.88E-05	25.97280527
351	389550	T	C	rs11648458	7.47E-06	0.0985747	0.022004	0.523282	5.32E-05	20.06898245
357	22255975	A	G	rs28791605	2.62E-06	0.178256	0.0379358	0.0842028	5.85E-05	22.07941271
3232	46291065	C	T	rs62073178	1.40E-09	-0.263708	0.0435526	0.0820856	9.72E-05	36.66199844
3288	15845369	G	A	rs12611282	1.35E-06	0.11039	0.0228444	0.358963	6.19E-05	23.35057893
3292	30491631	A	C	rs16964240	5.27E-06	-0.465772	0.102284	0.0158528	5.50E-05	20.7361939
3296	5695328	A	C	rs74963755	2.48E-06	0.199245	0.0423017	0.066964	5.88E-05	22.18487159
3305	27198147	T	G	rs77628790	8.17E-07	0.225982	0.0458256	0.0543639	6.45E-05	24.31806829

Supplementary Table 5. Summarized data of SNPs finally identified as IVs in our MR analyses（The causal relationship between PD and ever depressed for a whole week in East Asian ancestry）

SNP	chr	pos	other_allele	effect_allele	eaf	beta	se	*p*val	r2	F
rs11807932	1	206782184	G	C	0.021094451	1.504391256	0.315001219	1.79E-06	0.000129483	22.80826234
rs1433992	3	30278054	C	A	0.056782422	0.909526564	0.182943627	6.64E-07	0.000140316	24.71674483
rs278948	4	40464231	G	A	0.580104766	-0.37252221	0.082061585	5.64E-06	0.000116989	20.60722957
rs541615	9	27179530	A	G	0.441138887	-0.360929774	0.078013142	3.72E-06	0.000121515	21.40449306
rs646526	12	53065258	T	C	0.770459853	-0.462187041	0.093620457	7.94E-07	0.000138358	24.37188307
rs9375320	6	124641792	G	A	0.211410785	0.423791411	0.095869123	9.85E-06	0.000110935	19.54077844

Supplementary Table 6. Summarized data of SNPs finally identified as IVs in our MR analyses（The causal relationship between PD and MDD in East Asian ancestry）

SNP	chr	pos	other_allele	effect_allele	eaf	beta	se	*p*val	r2	F
rs11807932	1	206782184	G	C	0.021094451	1.504391256	0.315001219	1.79E-06	0.000129483	22.80826234
rs278948	4	40464231	G	A	0.580104766	-0.37252221	0.082061585	5.64E-06	0.000116989	20.60722957
rs541615	9	27179530	A	G	0.441138887	-0.360929774	0.078013142	3.72E-06	0.000121515	21.40449306
rs646526	12	53065258	T	C	0.770459853	-0.462187041	0.093620457	7.94E-07	0.000138358	24.37188307
rs9375320	6	124641792	G	A	0.211410785	0.423791411	0.095869123	9.85E-06	0.000110935	19.54077844

1.2 Supplementary Figures

Supplementary Figure 1. The causal relationship between PD and ever depressed for a whole week (PD from FinnGen r9 database).

(A) MR leave-one-out sensitivity analysis of PD's causal effect on having experienced depression for a whole week. The figure shows that excluding a particular SNP does not lead to a significant change in the overall result. (B) Scatter plot:The slope of the line corresponds to the causal estimate from each method. (C) The forest plot shows the estimate of the effect of genetically increased PD risk on ever depressed for a whole week risk. Each black dot represents the log odds ratio (OR) for ever depressed for a whole week per standard deviation (SD) increase in log OR for PD. Horizontal lines represent 95% confidence intervals (95% CIs). (D) Funnel plot showing the relationship between the causal effect of PD on ever depressed for a whole week estimated using each individual SNP as a separate instrument against the inverse of the standard error of the causal estimate. There is relatively symmetrical in the plot.

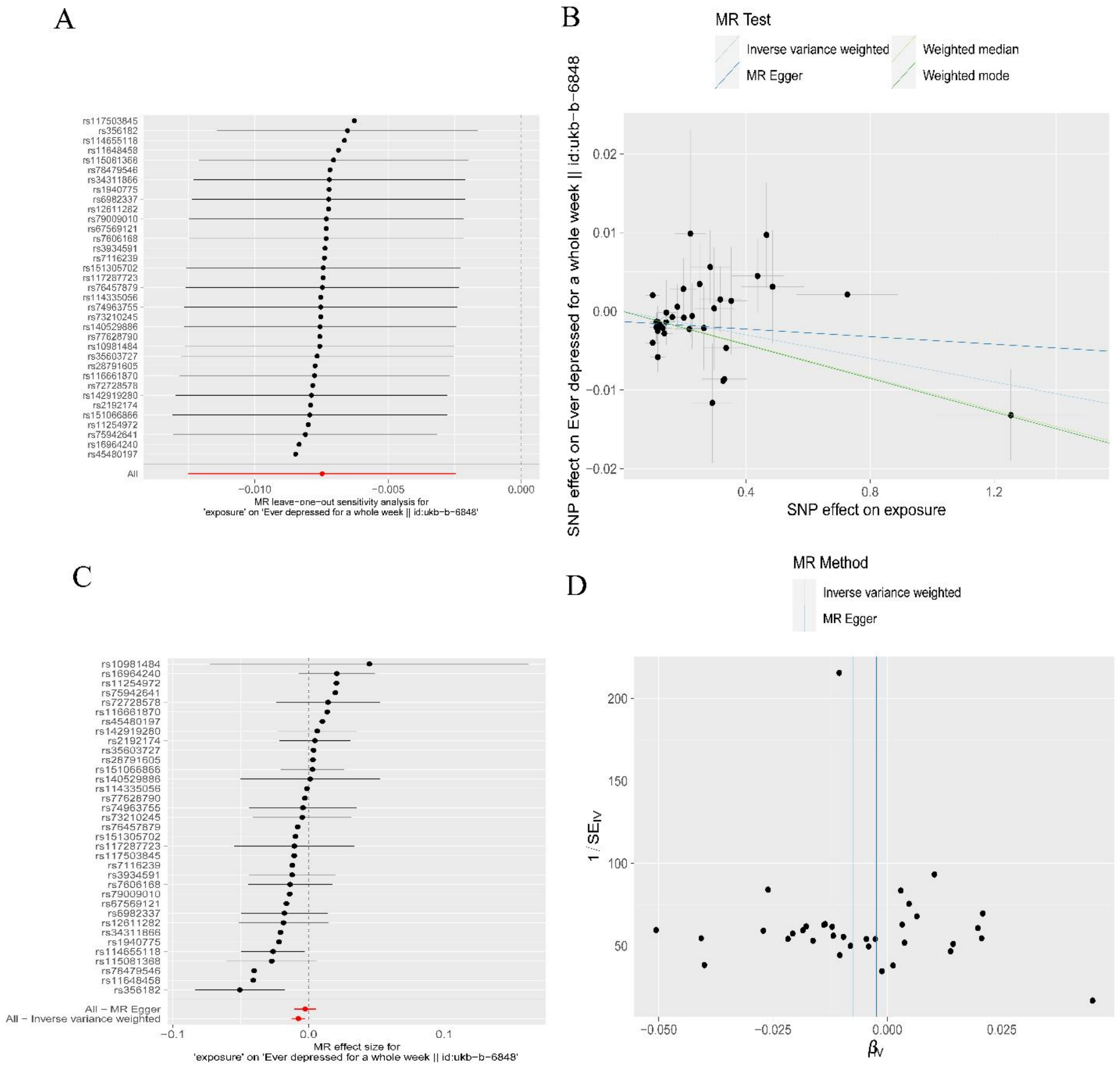
A
MR leave-one-out sensitivity analysis for
'exposure' on 'Ever depressed for a whole week || id:ukb-b-6848'
All
B
MR Test
Inverse variance weighted
MR Egger
Weighted median
Weighted mode
SNP effect on Ever depressed for a whole week || id:ukb-b-6848
SNP effect on exposure
C
All - MR Egger
All - Inverse variance weighted
MR effect size for
'exposure' on 'Ever depressed for a whole week || id:ukb-b-6848'
D
MR Method
Inverse variance weighted
MR Egger
1/SE_IV
β_IV

Supplementary Figure 2. The causal relationship between PD and MDD (PD from FinnGen r9 database).

(A) MR leave-one-out sensitivity analysis for PD on MDD. The figure shows that excluding a particular SNP does not lead to a significant change in the overall result. (B) Scatter plot:The slope of the line corresponds to the causal estimate from each method. (C) The forest plot shows the estimate of the effect of genetically increased PD risk on MDD risk. Each black dot represents the log odds ratio (OR) for MDD per standard deviation (SD) increase in log OR for PD. Horizontal lines represent 95% confidence intervals (95% CIs). (D) Funnel plot showing the relationship between the causal effect of PD on MDD estimated using each individual SNP as a separate instrument against the inverse of the standard error of the causal estimate. There is relatively symmetrical in the plot.

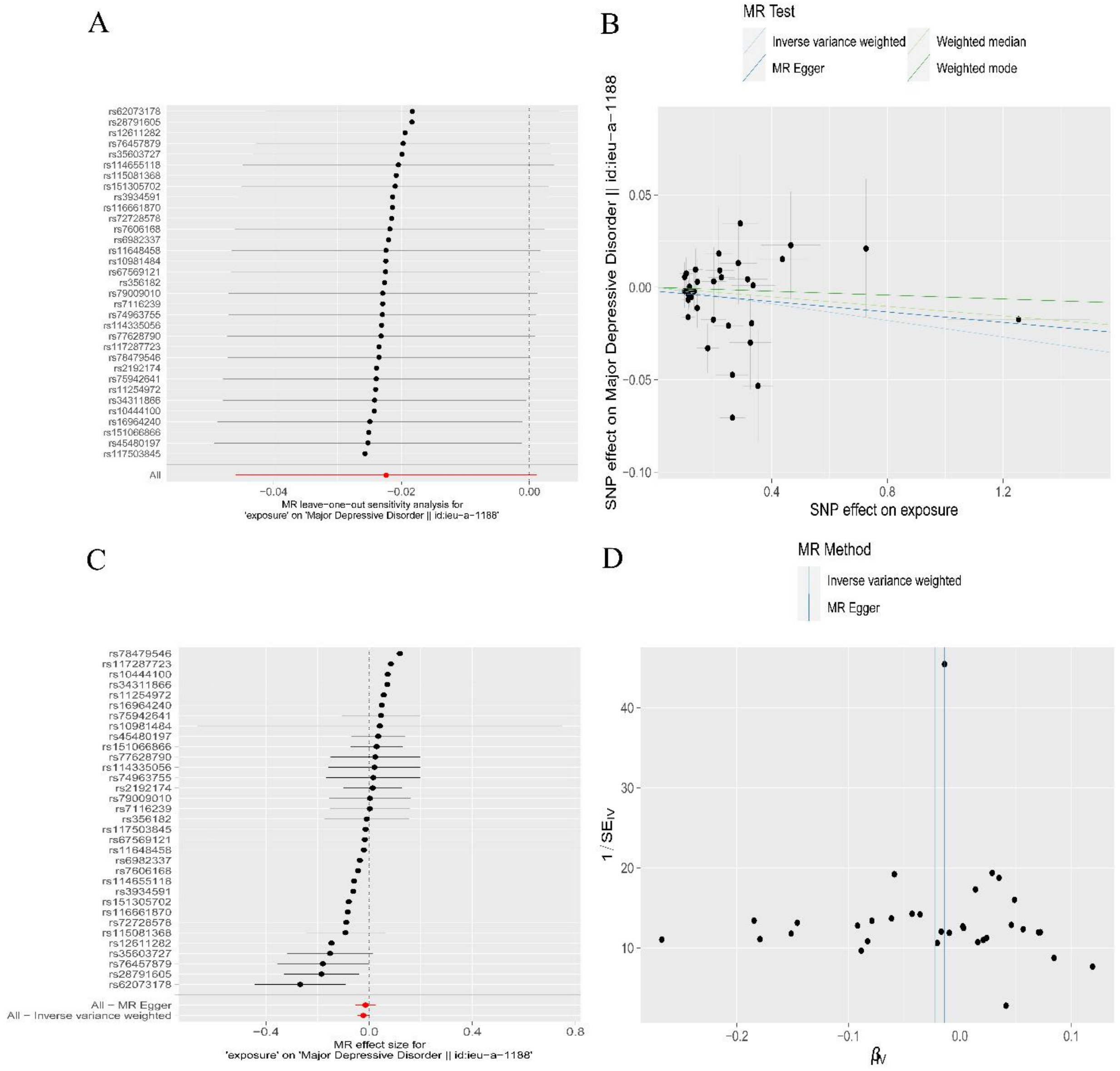
A
B
C
D
rs62073178
rs28791605
rs12611282
rs76457879
rs35603727
rs114655118
rs115081368
rs151305702
rs3934591
rs116661870
rs72728578
rs7606168
rs6982337
rs11648458
rs10981484
rs67569121
rs356182
rs79009010
rs7116239
rs74963755
rs114335056
rs77628790
rs117287723
rs78479546
rs2192174
rs75942641
rs11254972
rs34311866
rs10444100
rs16964240
rs151066866
rs45480197
rs117503845
All
−0.04
−0.02
0.00
MR leave−one−out sensitivity analysis for
'exposure' on 'Major Depressive Disorder || id:ieu−a−1188'
MR Test
Inverse variance weighted
MR Egger
Weighted median
Weighted mode
SNP effect on Major Depressive Disorder || id:ieu−a−1188
SNP effect on exposure
0.05
0.00
−0.05
−0.10
0.4
0.8
1.2
All − MR Egger
All − Inverse variance weighted
−0.4
0.0
0.4
0.8
MR effect size for
'exposure' on 'Major Depressive Disorder || id:ieu−a−1188'
MR Method
Inverse variance weighted
MR Egger
1/SEIV
βIV
40
30
20
10
−0.2
−0.1
0.0
0.1
